# A Group Parenting Intervention for Depressed Fathers (LTP + Dads): A Feasibility Study from Pakistan

**DOI:** 10.3390/children8010026

**Published:** 2021-01-06

**Authors:** Muhammad I. Husain, Imran B. Chaudhry, Ameer B. Khoso, Ming W. Wan, Tayyeba Kiran, Tinevimbo Shiri, Nasim Chaudhry, Nasir Mehmood, Syed F. Jafri, Farooq Naeem, Nusrat Husain

**Affiliations:** 1 Department of Psychiatry, University of Toronto, Toronto, ON M5S, Canada; farooq.naeem@camh.ca; 2 Department of Psychiatry, Ziauddin Hospital, Karachi 75600, Pakistan; ibchaudhry@btinternet.com; 3 Pakistan Institute of Living and Learning, Karachi 75600, Pakistan; ameer.bukhsh@pill.org.pk (A.B.K.); Tayyaba.kiran@pill.org.pk (T.K.); nasim.chaudhry@pill.org.pk (N.C.); 4 Division of Psychology and Mental Health, University of Manchester; Manchester M13 9PL, UK; m.w.wan@manchester.ac.uk (M.W.W.); nusrat.husain@manchester.ac.uk (N.H.); 5 Liverpool School of Tropical Medicine, Liverpool L3 5QA, UK; Tinevimbo.shiri@lstmed.ac.uk; 6 Karwan e Hayat, Karachi 75620, Pakistan; nasir_meh2003@yahoo.com; 7 Department of Community Health Sciences, Karachi Medical and Dental College, Karachi 74700, Pakistan; drfajafri2003@yahoo.com

**Keywords:** paternal depression, cultural adaptation, cognitive behavior therapy, parenting, low- and middle-income country

## Abstract

Background: Globally, paternal depression is a neglected and under-researched area. Aims: To feasibility test Learning Through Play Plus Dads (LTP+ Dads), a group parenting psychoeducation program adapted for depressed Pakistani fathers of children under 3 years of age. Methods: Fathers with depression were recruited in Karachi, Pakistan, for a pre-post feasibility study. Ten sessions of group LTP+ Dads were offered over three months. Clinical assessments were administered at baseline, three (end of intervention), and six (follow-up) months and included the Edinburgh Postnatal Depression Scale, 17-item Hamilton Depression Rating Scale, Brief Disability Questionnaire, Multidimensional Scale of Perceived Social Support, Euro-Qol-5 Dimensions, Rosenberg Self-esteem Scale, Parenting Stress Index, and Knowledge, Attitude and Practices questionnaire. Results: Of the 78 fathers approached, 34 consented to screening and 18 were eligible to participate. Participants had a mean age of 33 years, with a mean of 3.61 children. Most were unemployed and were from low-income households with low education backgrounds. The intervention was feasible and acceptable based on a recruitment rate of 100% of eligible participants and a 100% attendance rate for five of the 10 sessions. Fathers showed, on average, a reduction in depressive symptoms, an increase in most areas of knowledge, and positive attitudes about child development. Perceived social support, self-esteem, and functioning scores also increased. Conclusions: A low-cost, culturally adapted group intervention was found to be feasible and acceptable. Changes in depression, parenting-related, and other outcomes are promising and inform a future larger trial. Trial Registration: The trial was registered on Clinicaltrials.gov on 9 December 2020 (identifier: NCT04660253).

## 1. Introduction

Depression is the leading cause of disability worldwide [1] and low- and middle-income countries (LMICs) carry over 80% of this disease burden [2]. Parenting is a likely key mechanism for the intergenerational transmission of mental health risk [3], particularly in LMICs, as poverty undermines nurturing care in favor of survival. Researchers have tested parenting interventions for depressed mothers in LMICs, with positive outcomes for some parenting variables, while maternal depression has proved less amenable to change [4]. However, a parenting intervention in Pakistan that also targeted depression specifically benefitted both maternal mental health and parenting-related outcomes [5].

Globally, paternal depression is a neglected and under-researched area. In Pakistan, 23–42% of men are estimated to be depressed (based on the best available evidence) [6,7]. Pakistan is a patriarchal society and socioeconomic conditions may particularly undermine the father’s role. Poverty, low education, low social support, and family problems are risk factors for depression in Pakistan [8], and available treatments (i.e., antidepressant medication and cognitive behavior therapy (CBT)) are provided mainly through private healthcare in urban settings, which most families may not consider financially viable.

Given the high co-occurrence of paternal and maternal depression, paternal depression may respond better to treatment that incorporates family and social factors [9]. In a national Pakistani study, 36% of married women reported emotional abuse and 18% physical violence by their partner [10]. By providing cognitive and behavioral strategies for stress management and empowering fathers in the parental role through psychoeducation, an intervention for fathers may enhance the well-being of the family.

To our knowledge, there have been no studies on the prevalence of paternal depression in Pakistan. We report on a feasibility study of a culturally adapted, low-cost, psychosocial group intervention with a CBT component in a low-resource setting for depressed fathers with children under 3 years of age. The aim was to determine feasibility and acceptability, and report preliminary descriptive findings.

## 2. Methods

### 2.1. Participants

This pre-post design feasibility study was conducted between 1 August 2009 and 31 August 2010 in Karachi, Pakistan. Eighteen participants from a low-income area of Karachi were recruited via referral from a community health worker who approached potential participants in their home or community clinic. Potentially eligible fathers were provided information about the study and given 24 h to provide informed consent. All participants provided consent for their data to be used in the research.

Fathers with a child aged 0–3 years and who were depressed were eligible. Depression was determined by an Edinburgh Postnatal Depression Scale score >13 [11] and confirmed by interview using the Clinical Interview Schedule Revised (CIS-R) [12]. The CIS-R has been previously used in Pakistani studies [5]. Exclusions were any severe medical condition, significant physical or learning disability, current psychosis, or psychiatric care.

### 2.2. Intervention: Learning through Play+ Dads (LTP+ Dads)

Over a three-month period, fathers received a 10-session, low-cost, culturally adapted manualized group intervention deliverable by psychology graduates, which combines early child development information and group CBT, adapted for fathers. The intervention aimed to support fathers and equip them with the cognitive and behavioral tools and knowledge to be effective in their parental role as a pathway to alleviating paternal depression.

LTP [13] originated in Canada and has been culturally adapted for and use in LMICs. LTP+ [5] is structured around a calendar relatable to low-literacy parents to pictorially illustrate parent–child play and other activities that promote parental involvement, learning, and attachment. The intervention utilizes typical homemade toys, books, and materials accessible in low-resource settings. Sessions move in chronological order through the first 0–3 years of child development covering sense of self, physical, relationships, understanding of the world, and communication. Information is written in simple, low-literacy language. The CBT component helps parents identify and manage depression symptoms by addressing thoughts, moods, activities, and relationships [14].

LTP+ Dads is an adapted version that met a need voiced in the community following a previous randomized controlled trial (RCT) of LTP+ for depressed mothers [5] and (1) emphasizes the father’s role in child development and (2) coaches on strategies for managing stress, anger, conflict, time, nutrition, and safety [15]. While children do not attend sessions, fathers are encouraged to apply CBT and parent learning in the home and other contexts between sessions to promote generalization.

Our group has culturally adapted interventions for a range of adult mental disorders using mixed methods in Pakistan and the UK [16,17]. The framework for adaptation considers three areas: assessment and engagement, awareness of cultural factors, and adjustments in implementation. For LTP+ Dads, culturally acceptable idioms were used to explain the concept, causes, and symptoms of depression, taking into account lay perceptions and gender roles. Local folk stories and images (e.g., to explain the concept of multiple perspectives) and examples from religious teachings were also utilized. The intervention was delivered in Urdu, using culturally appropriate terms to establish rapport and trust.

### 2.3. Primary Feasibility Measures

Intervention feasibility was tested by collating data on recruitment and retention rates. The success criterion of feasibility was to recruit ≥50% of eligible fathers. Intervention acceptability was assessed using data on attendance. Criterion for acceptability was a mean attendance rate of ≥70% of at least five sessions.

## 3. Measures

A demographic questionnaire obtained personal information, including age, marital status, education, number of children in the household, employment, and loss of a child. All assessments were conducted at baseline, three months post-baseline (post-intervention), and six months post-baseline (follow-up). All measures were translated in Urdu and have been used in Pakistani samples [5]. For patient-rated measures, items were read to illiterate participants.

**Depressive symptom severity:** Edinburgh Postnatal Depression Scale (EPDS) [11] is a widely used, 10-item measure of postnatal depression that has been used with fathers in the postnatal period [18]. The 17-item Hamilton Depression Rating Scale (HAMD-17) [19] is the most widely used clinician-rated depression scale. In both scales, higher total scores reflect more severe depressive symptoms. In the current sample, Cronbach’s alpha for the EPDS andHAMD-17 were 0.536 and 0.419, respectively.

**Parenting-related measures:** Parenting Stress Index—Short Form (PSI) [20] Haskett et al., 2006) is a 36-item measure that yields a total stress score in the parenting role from three subscales (child difficulty, parental distress, and parent–child interaction). Higher scores indicate greater parenting stress. The Cronbach’s alpha for the PSI was 0.897. The Learning through Play Knowledge, Attitude and Practices (KAP) is a 114-item questionnaire [5] developed by the team to assess knowledge and attitudinal and behavioural change in five child areas (subscales) relevant to 0–3 years: physical development, sense of self, understanding about the world, relationships, and communication. Higher scores on the KAP scale indicate greater knowledge about parenting and more positive attitudes toward parenting.

**Resilience-related measures:** Rosenberg Self-Esteem Scale (SES) [21] is a 10-item scale of individual global self-worth. Multidimensional Scale of Perceived Social Support (MSPSS) [22] is a 12-item measure that assesses social support from family, friends, and significant others. Higher scores on the SES and MSPSS indicate greater self-esteem and perceived social support, respectively. In the current sample, Cronbach’s alpha for the SES and MSPSS were 0.623 and 0.956, respectively.

**Daily functioning measures:** Brief Disability Questionnaire (BDQ) [23] includes eight items about health problems that prevent carrying out daily activities. Euro-Qol-5 Dimensions (EQ-5D) [24] is a standardized measure of health based on five dimensions (mobility, self-care, usual activities, pain/discomfort, and anxiety/depression). In the current sample, Cronbach’s alpha for the BDQ and EQ-5D were 0.810 and 0.923, respectively.

## 4. Analysis

Participant characteristics and intervention outcomes were analyzed descriptively.

## 5. Ethical Approval

The Ethics Review Board (ERB) at the Pakistan Institute of Living and Learning provided approval for the study (ethics ref: 011/PILL/ERB/2009).

## 6. Results

**Figure 1:**
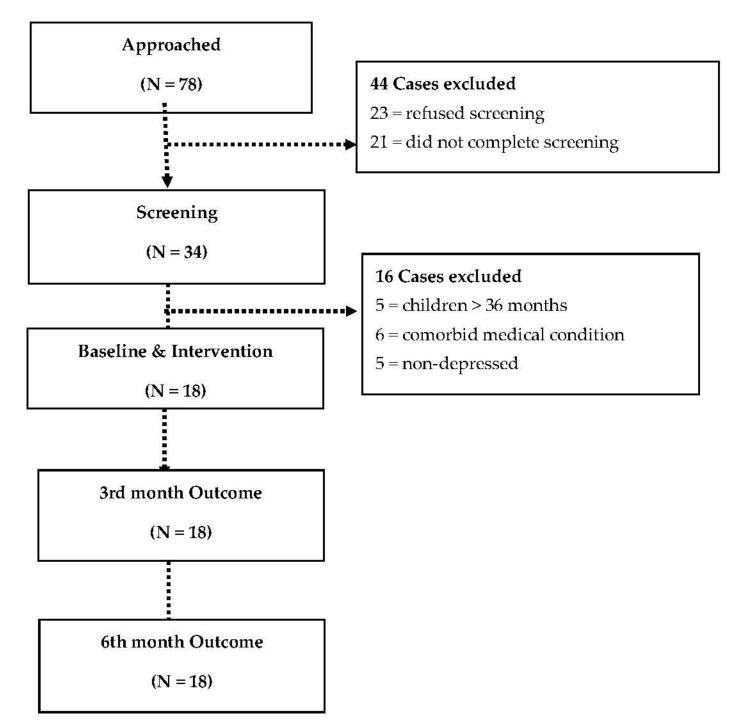
Consolidated Standards of Reporting Trials—CONSORT flow diagram.

Of the 78 fathers approached (see Figure 1 for Consolidated Standards of Reporting Trials - CONSORT diagram), 34 (43.59%) were screened, 23 (29.49%) refused screening, and we do not know the reasons but 21 (26.92%) did not complete the full EPDS. Of the 34 fathers who agreed to screening, five had children >3 years and six had a comorbid unstable medical condition such as tuberculosis/chronic obstructive pulmonary disease/heart failure. Of the remaining 23 who were screened, five did not meet the criteria for depression and 18 did. All 18 (100%) who met criteria (and had agreed to be screened) participated in the intervention and completed assessment at all time points, thus fulfilling our a priori feasibility criterion (of recruiting >50% of fathers screened and eligible for the study). All 18 (100%) participants attended at least five of the 10 sessions, which fulfilled our intervention acceptability criterion of 70% attending at least five sessions

Most participants were from low-income households with low education backgrounds and most were unemployed (Table 1). Paternal age varied widely. Seven participants (39%) had experienced the loss of a child.

Between baseline, post-intervention, and follow-up, depression symptomatology (EPDS and HAMD-17) and parenting stress (PSI) consistently reduced over time (Table 2). All five KAP subscale scores showed modest improvements at post-intervention or follow-up. Self-esteem (SES), perceived social support (MSPSS), disability (BDQ), and health (EQ-5D) scores also indicated improvements, with notable changes at three months in the last three outcomes.

## 7. Discussion

LTP+ Dads was shown to be feasible and acceptable by low-income depressed Pakistani fathers of a child 0–3 years, based on recruitment, attendance, and 100% retention. The fathers engaged with the study up to six months after baseline assessment and did not report any major difficulties in completing the outcome measures. To the best of our knowledge, this is the first study of paternal depression in Pakistan. In Pakistani culture, parenting styles differ greatly between genders. Societal expectation of paternal involvement in early childhood is generally low, as mothers tend to provide physical and emotional support. The level of uptake and retention was, therefore, encouraging, in that Pakistani fathers from a low-income background, generally with more traditional views and who were depressed, were open to participating in this parenting intervention in a group context.

Secondly, a lack of control group notwithstanding, our father outcome measures indicated that depression severity and parenting stress decreased following intervention and was sustained at follow-up, while most parenting KAP subscales improved at follow-up. The findings show promise for improving dysfunctional parent–infant interaction. Recognizing the father as a key influencer in the family potentially offers a systemic approach to breaking the intergenerational cycle of depression, as well as addressing paternal mental health. Furthermore, father perceptions of social support received from family and friends improved and were sustained, while self-esteem increased significantly at follow-up in many of the fathers who were unemployed.

These preliminary findings are similar to those found in an RCT of LTP+ for maternal depression conducted in an urban slum in Pakistan, which yielded improvements in maternal depression, parental stress, disability, and self-esteem, using identical outcome measures as the current feasibility study [5]. However, we reported substantially improved perceived social support in our father sample, which had not been found in the previous study with mothers. One explanation for this may be that, for many of the participants in the father sample, treatment goals focused on resolving interpersonal disputes, which is addressed in the CBT component of the intervention and led to improvement in social support networks.

## 8. Limitations

Although this study provides some insight into a relatively unexplored problem in Pakistan, this was a feasibility study with a small sample and results should be interpreted with caution. The results indicate some challenges with recruitment, with 43.59% of all fathers approached consenting to be screened for the study. Notwithstanding this, we were able to achieve the feasibility criteria for recruitment since 53% of those meeting eligibility criteria consented to study enrollment. The lack of a control group prevents any conclusions to be drawn regarding whether improvements were significantly better than routine treatment or whether fathers may have improved as a natural part of adjusting to the parental role or as children place relatively less demand on parents as they age. Further, child developmental outcomes were not assessed nor father–child interaction, so extension of assessment is needed to discern whether the intervention is beneficial to young children. Finally, our acceptability measures were relatively blunt; qualitative interviews would be valuable to gain nuanced information from fathers (and mothers) to inform refinement of the intervention.

## 9. Conclusions

To our knowledge, this study marks the first attempt to assess the engagement with a psychosocial parenting intervention for reducing paternal depression in fathers from a low-resource setting. The current feasibility findings inform and support the development of a larger scale RCT of LTP+ Dads in LMICs such as Pakistan, to confirm the current preliminary findings. Measuring child outcomes may strengthen the impetus for paternal depression interventions in LMIC settings, which bear the lion’s share of disease burden. By alleviating paternal depression in the child’s early years, a father may be in a unique position to influence maternal well-being and his child’s positive development.

## Figures and Tables

**Table 1:** Participant characteristics.

Personal and Household Characteristics	
	Mean (SD)
Father age	33.11 (8.49)
Monthly income (Pakistani rupees)	9083.33 (5018.35)
Number of children	3.61 (2.23)
	Frequency (%)
Household members	
Nuclear family	8 (44.44%)
Extended family (three or more generations in the same household)	10 (55.56%)
Educational level Below primary (Less than 5 years)	9 (50.00)
Primary to metric (up to 10 years of education)	7 (38.89)
Above metric (more than 10 years of education)	2 (11.11)
Current employment	5 (27.78)
Loss of a child	7 (38.89)
Financial debt	8 (44.44)

SD: Standard Deviation.

**Table 2:** Father depressive symptomatology, parenting-related outcomes, resilience outcomes, and physical functioning pre- and post-intervention and at follow-up (*N* = 18).

Outcome	Baseline (0 Month)	Post-Intervention (3 Months)	Follow-Up (6 Months)
	Mean [SD]	Mean [SD]	Mean [SD]
EPDS	14.17 [2.18]	7.00 [3.51]	6.06 [2.13]
HAM-D	16.44 [2.66]	11.33 [3.66]	9.89 [3.79]
PSI	91.17 [15.03]	79.67 [10.54]	73.61 [8.58]
KAP: Sense of Self	21.06 [3.21]	22.28 [1.67]	23.53 [1.94] ^a^
KAP: Physical Development	19.28 [1.41]	19.17 [1.58]	20.76 [1.75] ^a^
KAP: Relationships	21.06 [1.30]	21.11 [1.41]	22.76 [1.09] ^a^
KAP: Understanding of the World	16.83 [2.62]	18.22 [1.22]	19.41 [1.28] ^a^
KAP Communication	16.83 [1.62]	17.61 [1.20]	18.18 [1.07] ^a^
SES	18.33 [3.03]	19.78 [1.73]	20.28 [2.30]
MSPSS	34.28 [11.67]	56.89 [14.00]	55.00 [7.67]
BDQ	9.67 [4.42]	4.78 [2.65]	3.89 [1.45]
EQ-5D VAS score	40.28 [14.89]	59.17 [8.45]	59.44 [4.50]

BDQ = Brief Disability Questionnaire; EPDS = Edinburgh Postnatal Depression Scale; HAM-D = Hamilton Depression Rating Scale; EQ-5D VAS score = Euro-Qol-5 Dimensions visual analogue scale score; KAP = Learning Through Play Knowledge, Attitude and Practices questionnaire; MSPSS = Multidimensional Scale of Perceived Social Support; PSI = Parenting Stress Index; SES = Rosenberg Self-Esteem Scale. ^a^ 1 missing.

## Data Availability

Requests for sharing the anonymized trial database should be addressed to the lead author.
